# Ten-Gram-Scale Mechanochemical Synthesis of Ternary Lanthanum Coordination Polymers for Antibacterial and Antitumor Activities

**DOI:** 10.3389/fchem.2022.898324

**Published:** 2022-06-14

**Authors:** Liying Zhang, Haoran Shi, Xiao Tan, Zhenqi Jiang, Ping Wang, Jieling Qin

**Affiliations:** ^1^ School of Life Sciences and Medicine, Shandong University of Technology, Zibo, China; ^2^ Tongji University Cancer Center, Shanghai Tenth People’s Hospital, School of Medicine, Tongji University, Shanghai, China; ^3^ Institute of Engineering Medicine, Beijing Institute of Technology, Beijing, China

**Keywords:** lanthanum hybrids, solid-state reaction, coordination polymers, antibacterial performance, antitumor activities

## Abstract

As rare-earth coordination polymers (CPs) have appreciable antimicrobial properties, ternary lanthanum CPs have been widely synthesized and investigated in recent years. Here, we report convenient, solvent-free reactions between the lanthanum salt and two ligands at mild temperatures that form ternary lanthanum nanoscale CPs with 10-gram-scale. The structural features and morphologies were characterized using a scanning electron microscope (SEM), Fourier transform infrared spectrometer (FT-IR), ultraviolet-visible (UV–Vis), X-ray diffractometer (XRD), X-ray Photoelectron Spectroscopy (XPS), Brunauer–Emmett–Teller (BET), elemental analysis, inductively coupled plasma mass spectrometry (ICP-MS), electrospray ionization mass spectrometry (ESI-MS), nuclear magnetic resonance (NMR), dynamic light scattering (DLS) and analyzer, and thermogravimetric and differential thermal analyzer (TG-DTA). Furthermore, the *in vitro* antibacterial activities of these ternary hybrids were studied using the zone of inhibition (ZOI) method, minimum inhibitory concentration (MIC), minimum bactericidal concentration (MBC), and transmission electron microscope (TEM) and were found to have excellent antibacterial properties. The *in vitro* antitumor activities were performed in determining the absorbance values by CCK-8 (Cell Counting Kit-8) assay. This facile synthetic method would potentially enable the mass production of ternary lanthanum CPs at room temperature, which can be promising candidates as antibacterial compounds and antitumor agents.

## Introduction

Coordination polymers (CPs), which comprise organic and inorganic materials, act as linkers and nodes through the coordination interactions, respectively, and are promising carriers for use in several applications, including drug delivery (Giménez-Marqués et al., 2016; Wu et al., 2018; Ibrahim et al., 2020; Jiang et al., 2020), as sensors (Ma et al., 2013; Yao et al., 2016; Qin et al., 2019), and for catalysis (Li et al., 2016) owing to their diverse structures and highly enriched functions. Recently, the applications of CPs are mainly harnessed as a DNA molecular probe through fluorescence enhancement (Jin et al., 2017; Zhang et al., 2018), perovskite solar cell for the safe and flexible self-powered wristband system (Zhao et al., 2021), biodegradable nanozymes for the tumor-specific photo-enhanced catalytic therapy (Zhou et al., 2022), and a ultra-sensitive turn-on sensor for hydrogen sulfide gas detection (He et al., 2021). Over the past few decades, lanthanide complexes, a type of CPs, have been gaining increased attention in many technological applications, including catalysis (Furuno et al., 2000; Tosaki et al., 2005), magnetic resonance imaging (MRI) (Wang et al., 1992; Andolina et al., 2012), and in the manufacture of bactericide (Karthikeyan et al., 2004; Kaczmarek et al., 2018; Busila et al., 2020). Among them, the antibacterial performance of the CPs has been primarily focused on owing to their outstanding ability as antimicrobial agents *in vitro* (Zhao et al., 2012; Zhou et al., 2020).

Several studies have reported that 8-hq can be used as topical antiseptics, disinfectants, and anti-inflammatory agents because of its high lipophilicity to penetrate bacterial cell membranes (Andreeva et al., 2012). However, pure 8-hq may not be clinically applied, owing to its toxic nature and side effects and especially carcinogenicity in antitumor (Nan et al., 2013). In 2017, 8-hq is classified as a carcinogen by the World Health Organization’s International Center for Research on Cancer (Chan et al., 2013). Meanwhile, the 5-SSA itself has weak antibacterial properties and is often used in combination with metal compounds for their biological activities, such as antimicrobial, antifungal, anti-inflammatory, analgesic, anthelmintic, antiulcer, antitumor, and carbonic anhydrase inhibitor properties (Ilkimen et al., 2018). As rare-earth complexes obtain fewer toxic substances than many organic synthetic substances or excessive metal complexes, the preparation of rare-earth–based CPs can not only attenuate the toxicity of rare-earth complexes but also enhance their antitumor activity and stability simultaneously (Taha et al., 2016).

Ternary CPs are usually prepared using the liquid phase method (Gu et al., 2015; Den Auwer et al., 2000), through which the final products may contain impurities owing to the presence of the solvent. Furthermore, the liquid phase method also has limitations such as excessive energy consumption and low yield, which hinder mass production (Feng et al., 2020). In that case, several solvent-free methods, such as gas phase method, high temperature, grind, and microfluid, were harnessed instead of the solution phase method for the synthesis of CPs efficiently (Zhou et al., 2013; Li et al., 1998; Min et al., 2011). For instance, J. Nathan Hohman and his laboratory team prepared a kind of CP, silver benzeneselenolate, with the gas phase method. The large-area synthesis of the hybrid chalcogenide thin films in mild temperature is a useful method for the application of CPs in hierarchical architectures and semiconductor devices (Trang et al., 2018). Ivan Halasz et al. explored the CP with mechanochemical milling reactions through which the ideal solvent-free environment can provide a diversity of transformations (Halasz et al., 2013). Jonathan W. Steed et al. synthesized the CP metal tris(phenanthroline) cations into p-sulfonatocalix (Ibrahim et al., 2020) arene anions by manual grinding based on the charge-assisted π-stacking interactions between polymers and metals (Nichols et al., 2001). Compared with the solvent-phase methods, solvent-free methods can be performed at room temperature (RT) to yield multiple functional materials and obtain convenient synthetic procedure with less environmental contamination (Li et al., 2007; Cao et al., 2009).

In this work, a kind of solvent-free method for a greener mechanochemical preparation of lanthanum CPs with 10-gram-scale at RT was introduced, and its potential use as an excellent antimicrobial and antineoplastic material has been proposed. The morphologies of the compounds and the *in vitro* antibacterial and antitumor experiments were characterized afterward. Compared with the CPs prepared using a solution-phase reaction, the mechanochemically synthesized lanthanum CPs obtained relatively high antibacterial activity and a strong inhibitory effect on tumor proliferation.

## Materials

The materials, the characterization, and the determination of antibacterial and antitumor activities were specified in Supplementary Material.

### Cell Lines

The human colorectal cancer cells HCT116, human breast cancer cells MCF7, and human bladder cancer cells T24 were received from the National Collection of Authenticated Cell Cultures.

The HCT116 and MCF7 cells were grown in DMEM (Wisent Inc.) supplemented with 10% fetal bovine serum (ExCell Bio) and 1% penicillin–streptomycin solution (10,000 IU·mL^−1^ penicillin and 10 mg mL^−1^ streptomycin) in a humidified atmosphere of 5% CO_2_ at 37°C. The T24 cells were grown in RPMI 1640 (Wisent Inc.) medium supplemented with 10% fetal bovine serum (ExCell Bio) and 1% penicillin–streptomycin solution (10,000 IU·mL^−1^ penicillin and 10 mg mL^−1^ streptomycin) in a humidified atmosphere with 5% CO_2_ at 37°C.

### Preparation of Ternary CPs in Mechanochemical Method

**FIGURE 1:**
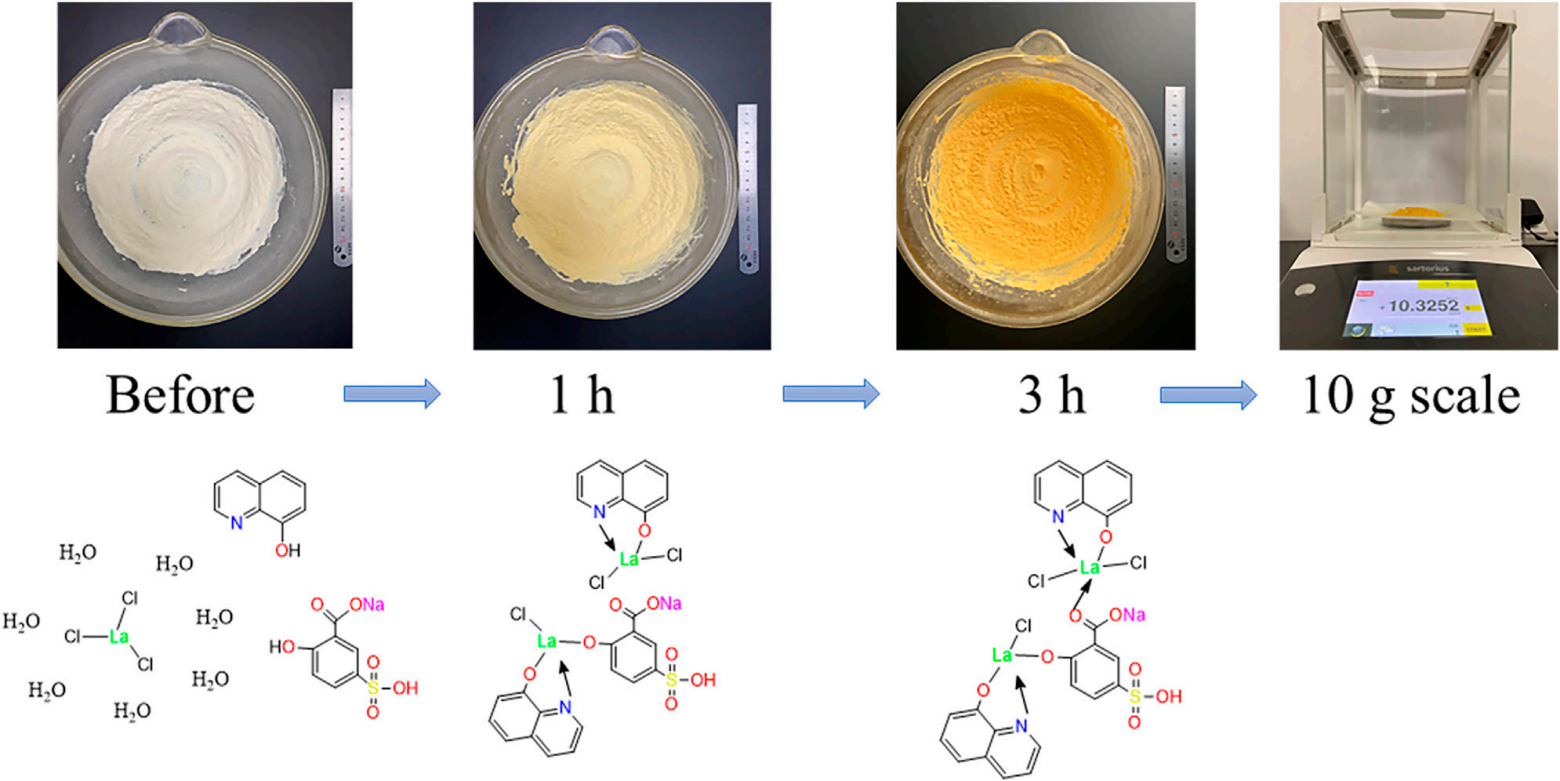
Preparation of 10-gram-scale ternary lanthanum CPs and its possible coordination bonds in the structure

For the synthesis of solvent-free CPs, appropriate quantities of 5-sulfosalicylic acid sodium salt (5-SSA) and 8-hydroxyquinoline (8-hq) were weighed as shown in Supplementary Table S1 and ground in an agate mortar to receive a uniform powder. The two ligands were then mixed together with further pulverization for 30 min. After that, LaCl_3_·7H_2_O was added with continuous pulverization for another 3 h. The successful coordination of lanthanide ions and ligands was formed when the color of the complex gradually changed from white to pale yellow and finally changed to dark yellow in Figure 1. The mixture was continued to be ground at 25°C for 1 h to obtain the well-prepared products. Last, the precipitate was washed with distilled water (DW)/ absolute ethanol (EtOH) three times in succession and then dried in a vacuum oven to obtain the 10-gram scale. The yellow product prepared with manual grinding using different reactant ratios was investigated (Supplementary Table S1). In addition, the ternary lanthanum CPs were also prepared through solution phase for comparison. After the successful preparation, the structure and chemical components were determined by the elemental analysis, ICP-MS, ESI-MS (Supplementary Figure S1), and NMR (Supplementary Figures S2, 3). The elemental analysis found (%) C 35.28, H 1.9, N 3.4, S 3.15, and O 12.54; ICP-MS: 27.81% La. As shown in the ESI-MS, the molecular weight of [MS-2H^+^] appeared to be 912.6347 g/mol, indicating the successful synthesis of LCP. Moreover, the ^1^H NMR values are as follows: (600 MHz, DMSO) δ 13.95 (s, 1H), 9.84 (s, 1H), 8.83 (s, 2H), 8.30 (s, 2H), 8.15 (s, 1H), 7.60–6.90 (m, 9H), and 6.70 (s, 1H) and ^13^C NMR values are as follows: (151 MHz, DMSO) δ 175.56, 167.03, 162.19, 148.19, 138.73, 136.77, 131.09, 129.59, 128.42, 122.07, 117.38, 115.67, and 111.83. According to the aforementioned results, two ternary lanthanide complexes are suggested to have a general chemical formula of La_2_Cl_3_(5-SSA)_1_ (8-hq)_2_ (Solórzano et al., 2018; Suárez-García et al., 2021). The possible coordination bonds of the final CPs are illustrated in Figure 1.

### CCK-8 Cell Viability Assay

The cytotoxicities of rare-earth complexes and of three ligands (8-hq, LaCl_3_·7H_2_O, and 5-SSA) toward three tumor cells (HCT116, MCF7, and T24) were tested by the CCK-8 (Cell Counting Kit-8) assay. The cells were seeded in 96-well plates at a density of 5,000 cells per well, and the samples were added to the wells (100 µL per well) at different concentrations (0 μg/ml, 0.3 μg/ml, 0.6 μg/ml, 1.25 μg/ml, 2.5 μg/ml, 5 μg/ml, 10 μg/ml, 20 μg/ml, and 40 μg/ml) after 24-h incubation. After further culture at 37°C for 48 h, 10 µL of CCK-8 (Beyotime Biotechnology) solution was added to each well and incubated for another 2 h followed by the absorbance recording at 450 nm using BioTek 800 TS Absorbance Reader. The cell survival rate was calculated using the following formula: survival rate (%) = [(As-Ab)/ (Ac-Ab)] × 100%, where As represents the mean absorbance of test wells, Ac represents the mean absorbance of control wells, and Ab represents the mean absorbance of blank wells.

The IC_50_ values of LCP-1 and of three ligands (8-hq, LaCl_3_·7H_2_O, and 5-SSA) toward three tumor cells (HCT116, MCF7, and T24) were calculated by using the lg(inhibitor) *vs*. response-variable slope curve fitting equation of GraphPad Prism 8 software (Graph Pad Software Inc., United States). All data were expressed as the mean ± SD.

## Results and Discussion

### Characterization of the Ternary Lanthanum CPs

**FIGURE 2:**
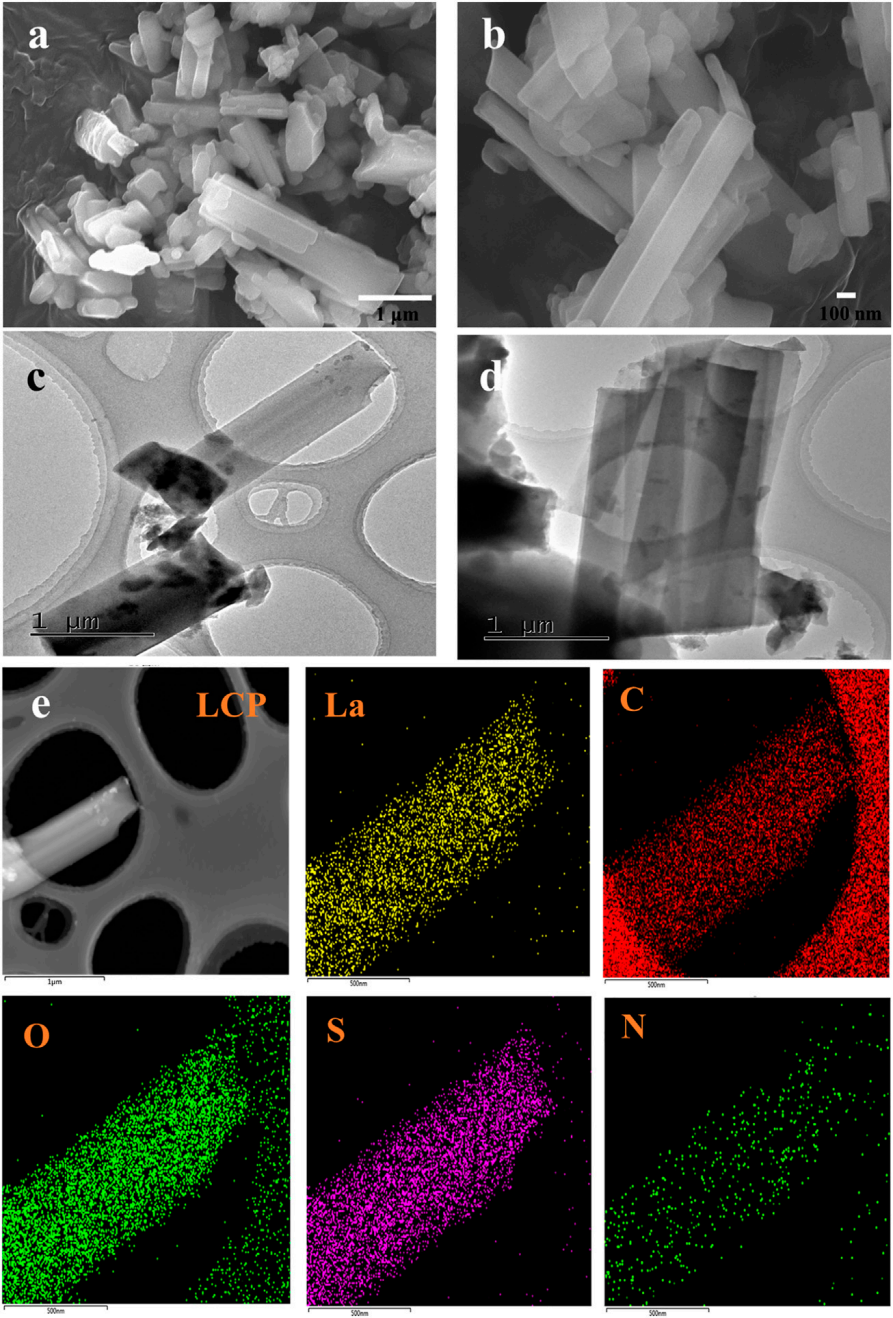
SEM **(A,B)** and TEM **(C,D)** images of LCP-1 with different resolutions, **(E)** STEM elemental mapping of La, C, O, S, and N for LCP-1.

The morphologies of the ternary lanthanum CPs, namely, LCP-1, LCP-2, and LCP-3 were evaluated using SEM, TEM, DLS, FT-IR, UV-Vis, XRD, BET surface, and TG-DTA. As shown in Figure 2, the SEM and TEM images in (a–d) reveal LCP-1 to have a rod-shaped, composite structure. In addition, the result of the STEM mapping (Figure 2E) shows a successful preparation of the coordination polymer with nanostructure using LaCl_3_·7H_2_O, 5-SSA, and 8-hq. The LCP-1 for DLS was appropriately diluted and measured in water with a hydrodynamic median diameter of median diameter 6.78 μm (Supplementary Figure S4). The LCP-2 reactants in Supplementary Figures S5A,B using a similar method appear to comprise short rods and cubes. Interestingly, Supplementary Figures S5C,D shows the solution-state products of LCP-3 as piled layers with a spindle structure, which differ from the rod- or cube-like forms of the solid-state hybrids. Different methods result in nanocomposites with diverse morphologies, which may affect the antibacterial activity.

**FIGURE 3:**
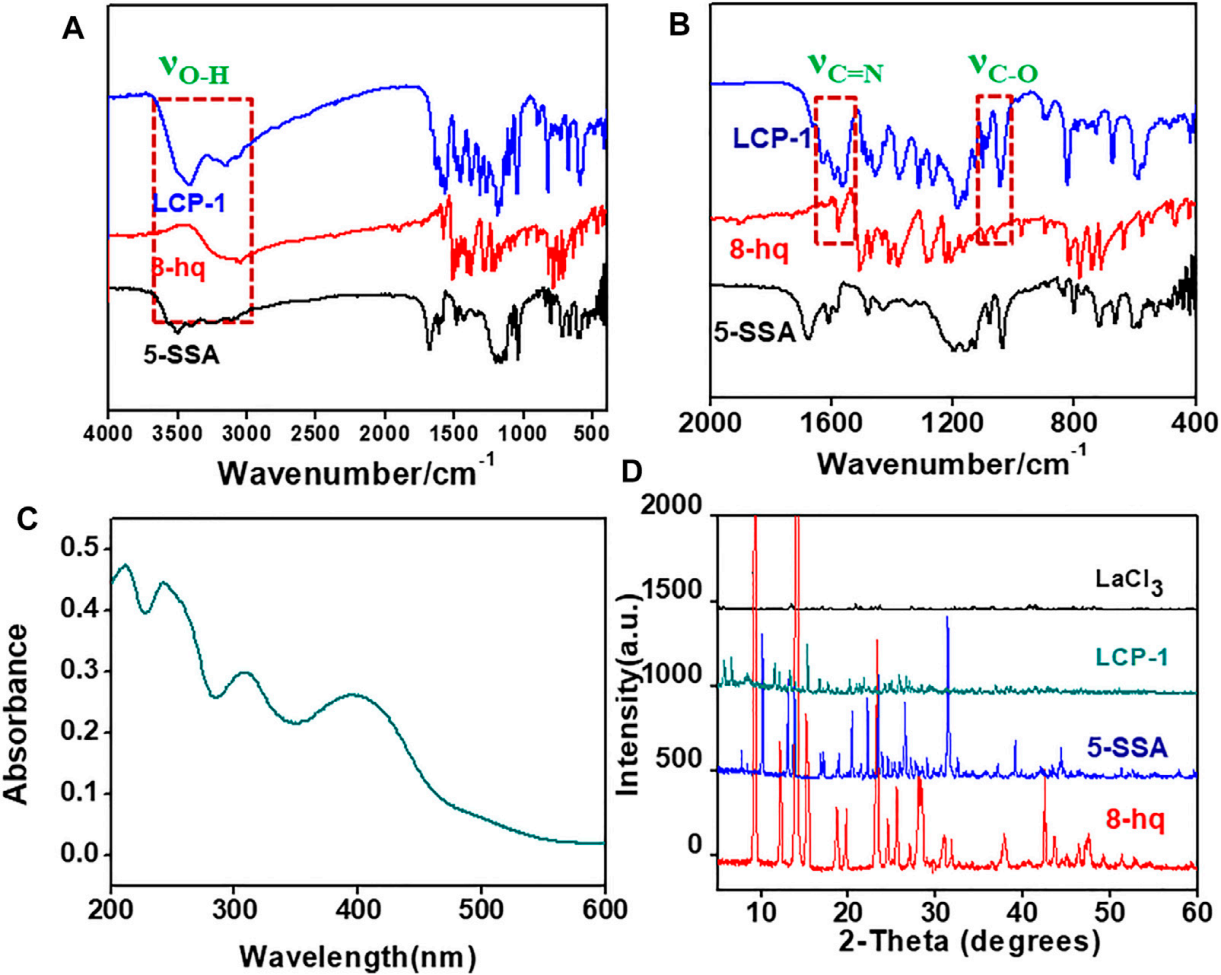
FT-IR spectra of CP-1 and the reactants **(A)**, the amplification from 2000 cm^−1^ to 400 cm^−1^ **(B)**, UV–Vis spectra of LCP-1 **(C)**, and XRD patterns of LCP-1**(D)**.

After product characterization using SEM, the functional groups of the ternary lanthanum CPs were investigated using FT-IR and summarized in Supplementary Table S2 (Feng et al., 2001; Zheng et al., 2015). As shown in Figures 3A,B, the peaks at 3,051 cm^−1^ and 1,223 cm^−1^ are assigned to O-H bending, whereas the other two peaks at 1,093 cm^−1^ and 1,579 cm^−1^ represent the stretching vibrations of C-O and C=N in 8-hq (Shen et al., 1994; Gavrilko et al., 2004; Obot et al., 2017). Compared to the ternary lanthanum complex, the stretching (*ν*_O-H_, 8-hq, 3,051 cm^−1^) and bending vibrations (*δ*_O-H_, 8-hq, 1,223 cm^−1^) of LCP-1 disappears, while the ν_C-O_ peak at 1,093 cm^−1^ in 8-hq is red shifted about 10 cm^−1^, indicating the formation of the La-O bond between the ligand and La ions. This occurrence is because the electronegativity of La ions is less than that of oxygen, and the increase in the electronegativity of oxygen can enhance the stretching vibrations of C-O. The *ν*_C=N_ is blue shifted to 1,591 cm^−1^, demonstrating that the La ions are ligated with the hydroxyl oxygen and hetero-nitrogen atoms of 8-hq forming a five-membered chelating ring. On the other hand, the broad peaks of 5-SSA from 3,500 cm^−1^ to 3,200 cm^−1^ are attributed to O-H and C-H bending, while the peaks between 1,300 cm^−1^-1,000 cm^−1^ indicate the specific peaks of SO_3_^−^. The peaks at 1,430 cm^−1^ and 1,678 cm^−1^ are assigned to the symmetric and asymmetric stretching vibrations of COO, respectively (Jiang et al., 2002; Lu et al., 2006). As shown in Figure 3B, the sharp peaks of the COO group in LCP-1 is blue shifted from 1,430 cm^−1^ and 1,678 cm^−1^ to 1,313 cm^−1^ and 1,558 cm^−1^ separately, indicating the chelation of La ion with both the ligands, 5-SSA and 8-hq. In addition, the gap between the symmetric (*ν*_s_, 1,313 cm^−1^) and asymmetric stretching (*ν*_as_, 1,558 cm^−1^) vibrations of the COO group (△*ν* = 248 cm^−1^, 5-SSA △*ν* = 245 cm^−1^, LCP-1) proves the bidentate chelating coordination of the La ion with 5-SSA.

Compared to LCP-1 and LCP-2 in Supplementary Figures S6A, no evidence could be found for the shifts or changes. Therefore, it can be deduced that the same method can obtain similar ternary lanthanum CPs even with different molar ratios of the reactants. However, the compound LCP-3 prepared using the solution phase method showed a significant difference in the broad peaks between 800 cm^−1^−600 cm^−1^, 1,200 cm^−1^−1,000 cm^−1^, and 1,600 cm^−1^−1,400 cm^−1^, indicating diverse nanocomposites even when using a similar composition.

The UV spectrum in Figure 3C can reflect the electron transition produced by the absorption of energy and mainly reflects the properties of the unsaturated groups in the molecule (Hassaan and Khalifa, 1996; Qiang-Guo et al., 2001). The interactions between the solvent and solute may affect the molecular energy levels, and consequently, the shape of the UV spectrum, the location of the absorption band, and the intensity. Figure 3C and Supplementary Figures S6B reveal similar UV–Vis spectra for LCP-1 and LCP-2 at 212, 243, 308, and 400 nm, revealing the similarity in composition and structure of the ternary lanthanum complexes. Different spectra of LCP-3 show peaks at 229, 267, and 337 nm, suggesting that different synthetic methods result in distinctly dissimilar ternary lanthanum samples.

The lanthanide chloride hydrates, 8-hq and 5-SSA, and the as-prepared ternary lanthanide CPs were detected in the XRD patterns as seen in Figure 3D (Min et al., 2011). The product peaks are visibly different from those of the reactants, indicating the successful preparation of the ternary lanthanide complex. The similarity in the XRD patterns of LCP-1 and LCP-2 (Supplementary Figures S6C), and that in the different peaks of LCP prepared in the mechanochemical and solvent methods suggests that the same method could be used in the preparation of the same product and vice versa, which is in agreement with the results of UV–Vis and IR spectroscopy.

**FIGURE 4:**
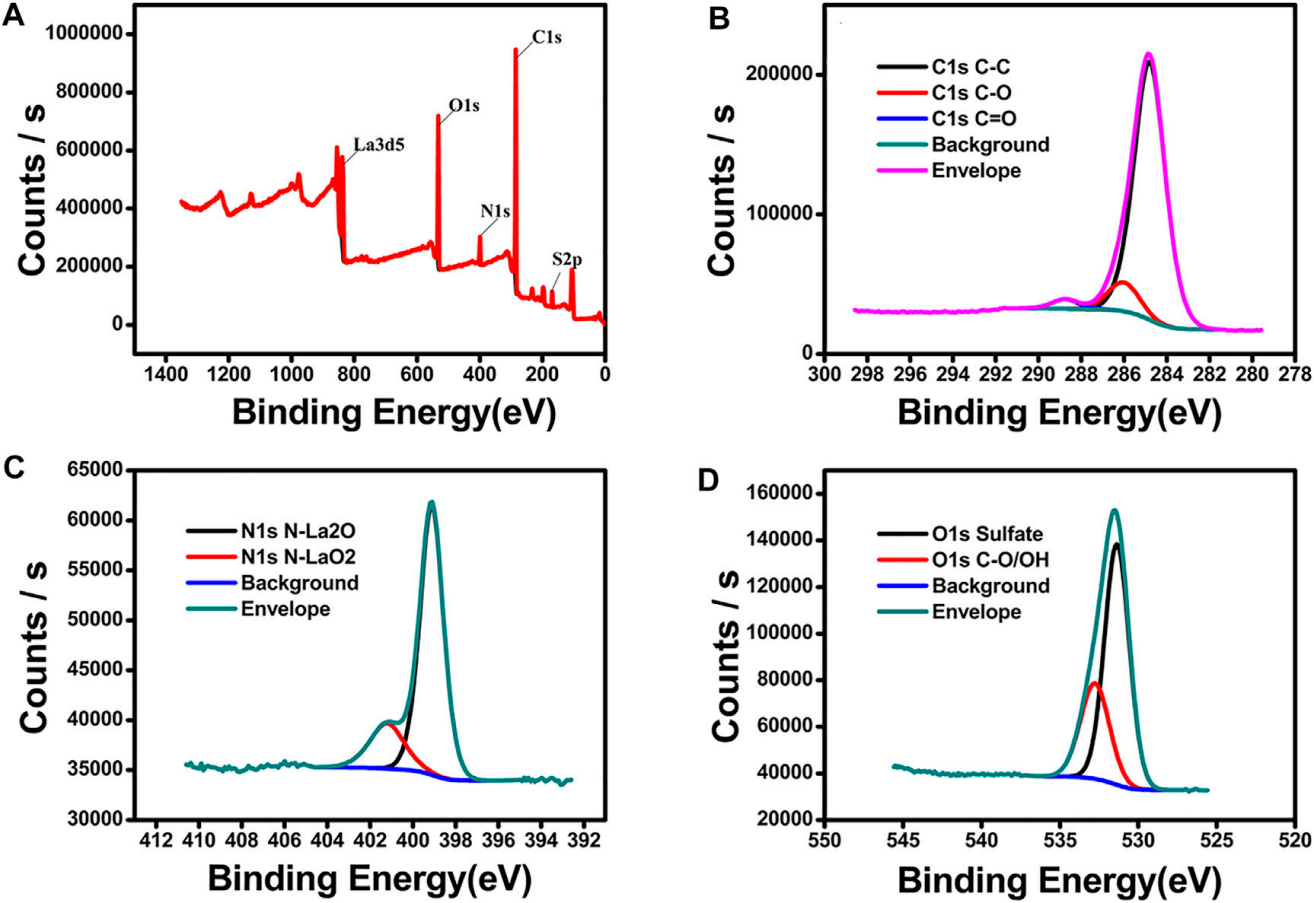
Survey-scan XPS spectrum of LCP-1 **(A)** and high-resolution spectra of C1s **(B)**, N1s **(C)**, and O1s **(D)**.

Figure 4 shows the XPS survey spectra of LCP-1, revealing the presence of the carbon, nitrogen, oxygen, sulfur, and lanthanum elements (Figure 4A). No other elemental contaminants were detected. The C1s signal in Figure 4B was deconvoluted with contributions corresponding to the well-identified sulfur bonds present in LCP1: at 288.74 eV assigned to C=O, at 284.8 eV assigned to C-C, and at 286.02 eV assigned to C-O. The peaks associated with N1s in Figure 4C are centered at around 399.11 and 401.2 eV, which are assigned to the signals of N-La2O and N-LaO2, respectively. The O1s core level spectra of LCP1 showed two characteristic deconvoluted peaks (Figure 4D). The first component centered at 531.36 eV was assigned to the O-S bond, and the second component centered at 532.76 eV was assigned to the C-O/O-H bond. In summary, the XPS results further confirmed the successful preparation of LCP-1.

In addition, N_2_ adsorption–desorption isotherms were utilized to measure the BET of LCP-1 in Supplementary Figure S7. The lack of hysteresis (P/P_0_ < 0.9) indicates that LCP-1 has a typical mesoporous structure with the BET surface areas of 5.5797 m^2^/g.

**FIGURE 5:**
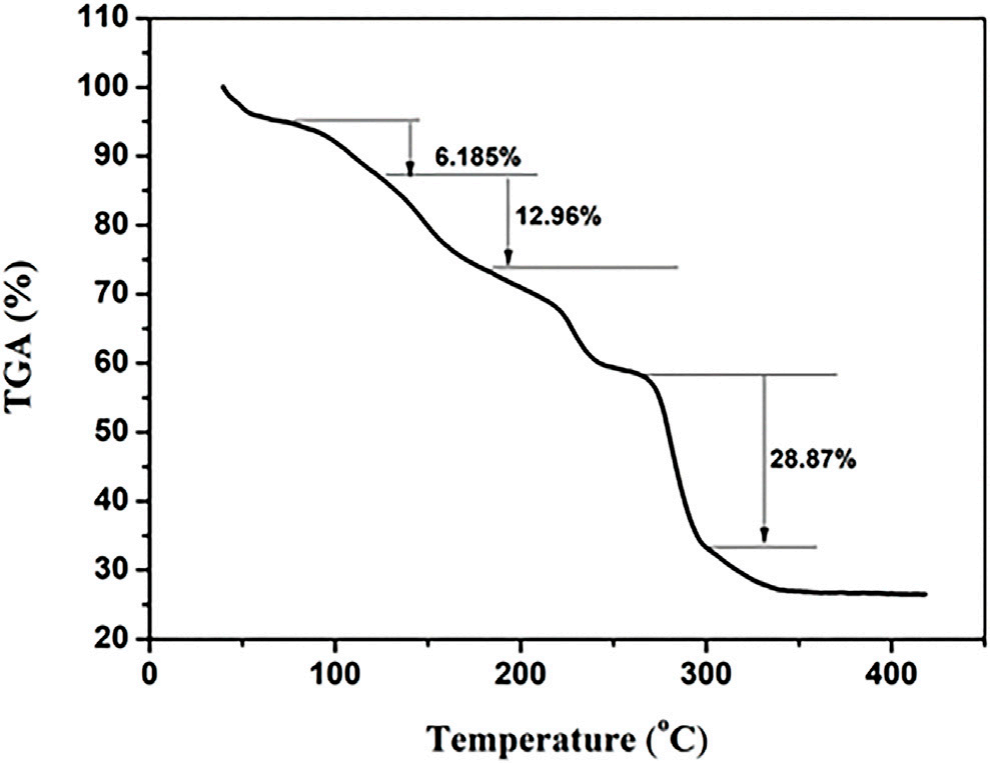
Differential heat–heat weight map of LCP1.

The thermal analysis result is shown in Figure 5, which was consistent with that in our previous study (Wang et al., 2021). The first minor weight loss (6.185%) occurred at RT to about 100°C, indicating solvent and water loss in LCP1, while the two stage weight loss (12.96%, 28.87%), with the increase of the temperature from 200°C–400°C, corresponds to the fracture of the functional groups and the main structure of LCP, respectively. The remaining 26.11% weight corresponds to lanthanum oxide.

### Antibacterial Activity of the Ternary Lanthanum CPs

After the successful preparation and characterization of the ternary lanthanum CPs, the *in vitro* cumulative release of LCP1 with time was evaluated by ICP-OES with the maximum release, which was about 15% at 24 h, indicating its relative stability in PBS (pH 7.4) (Supplementary Figure S8). The *in vitro* antibacterial activities were investigated afterward using the zone of the inhibition method and by determining the MIC and MBC values.

Table 1 shows the ZOI of the synthesized CPs against *E. coli*, *S. aureus*, *S. typhi*, and *P. aeruginosa*. Compared to 8-hq, the inhibition diameters of LaCl_3_ and 5-SSA are relatively smaller. However, the antibacterial activity of the nanoproducts, in Table 1 and Supplementary Table S3, is excellent owing to the antibacterial synergy effect. The antibacterial effects of lanthanum CPs are similar owing to their similarity with the coordination structure of 8-hq. On the other hand, the ZOI of all samples against *S. aureus* is the strongest compared to that observed in the other three bacteria. A possible reason is that although the outer membrane of Gram-positive bacteria is much thicker than that of Gram-negative bacteria, due to the loose membrane of the Gram-positive bacteria, the antibacterial agent can damage the membrane, enter the bacterial cell, and gradually cause a bactericidal effect (Yang et al., 2014; Yang et al., 2015).

**TABLE 1:** ZOI, MIC, and MBC of the composites and their reactants.

Sample	Inhibition Zone (mm)/ MIC/ MBC			
	*E. coli*	*S. aureus*	*S. typhi*	*P. aeruginosa*
LaCl_3_	8/ >800/ >800	9/ >800/ >800	8/ >800/ >800	8/ >800/ >800
8-hq	25/100/ 100	27/ 50/ 100	24/ 100/ 200	23/100/ 100
5-SSA	7/ >800/ >800	8/ >800/ >800	7/ >800/ >800	8/ >800/ >800
LCP-1	23/200/ 400	25/ 100/ 200	22/ 200/ 400	21/200/ 200

The MIC/MBC is defined as the lowest concentrations of the sample which prevents visible growth or kills bacteria. Generally, the MIC values of the ternary lanthanide against the tested bacteria ranged from 50–200 ppm, while the MBC values ranged from 100–400 ppm.

Based on the results presented in Table 1, 8-hq shows the highest antibacterial activity, while the ternary composites have similar MIC and MBC values. In addition, the MIC/MBC results of *S. aureus*, the Gram-positive bacterium, are the best compared to those of the Gram-negative bacteria, and consistent with the results obtained from the determination of the ZOI.

**FIGURE 6:**
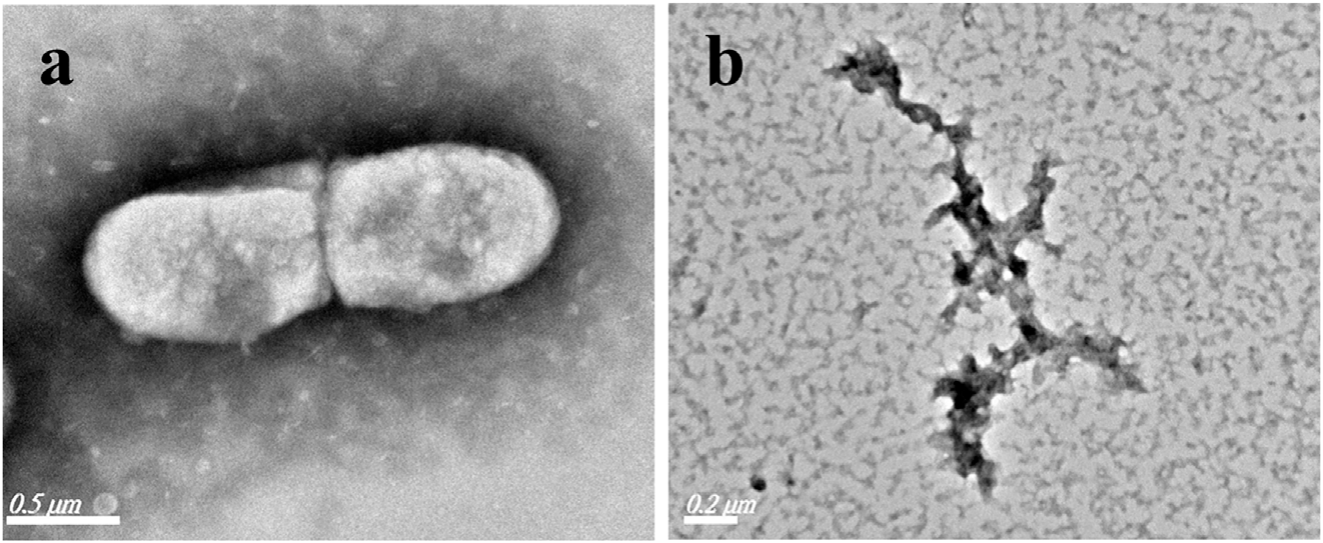
TEM images of *E. coli* before **(A)**/ after **(B)** treatment with LCPs

For further understanding the mechanism of the bactericidal effect, the morphological changes of the typical Gram-negative bacterium *E. coli* were investigated by TEM before/after the addition of LCPs. As shown in Figure 6, the original *E. coli* obtains a rod-like structure with the size of 1 µm*0.5 µm in the absence of LCPs. However, after the treatment of LCPs, the bactericide interacts with the membrane, making the cytoplasm to flow out and decompose the bacteria. The TEM results are also consistent with the results received from the other bactericidal experiments.

### Antitumor Activities

**FIGURE 7:**
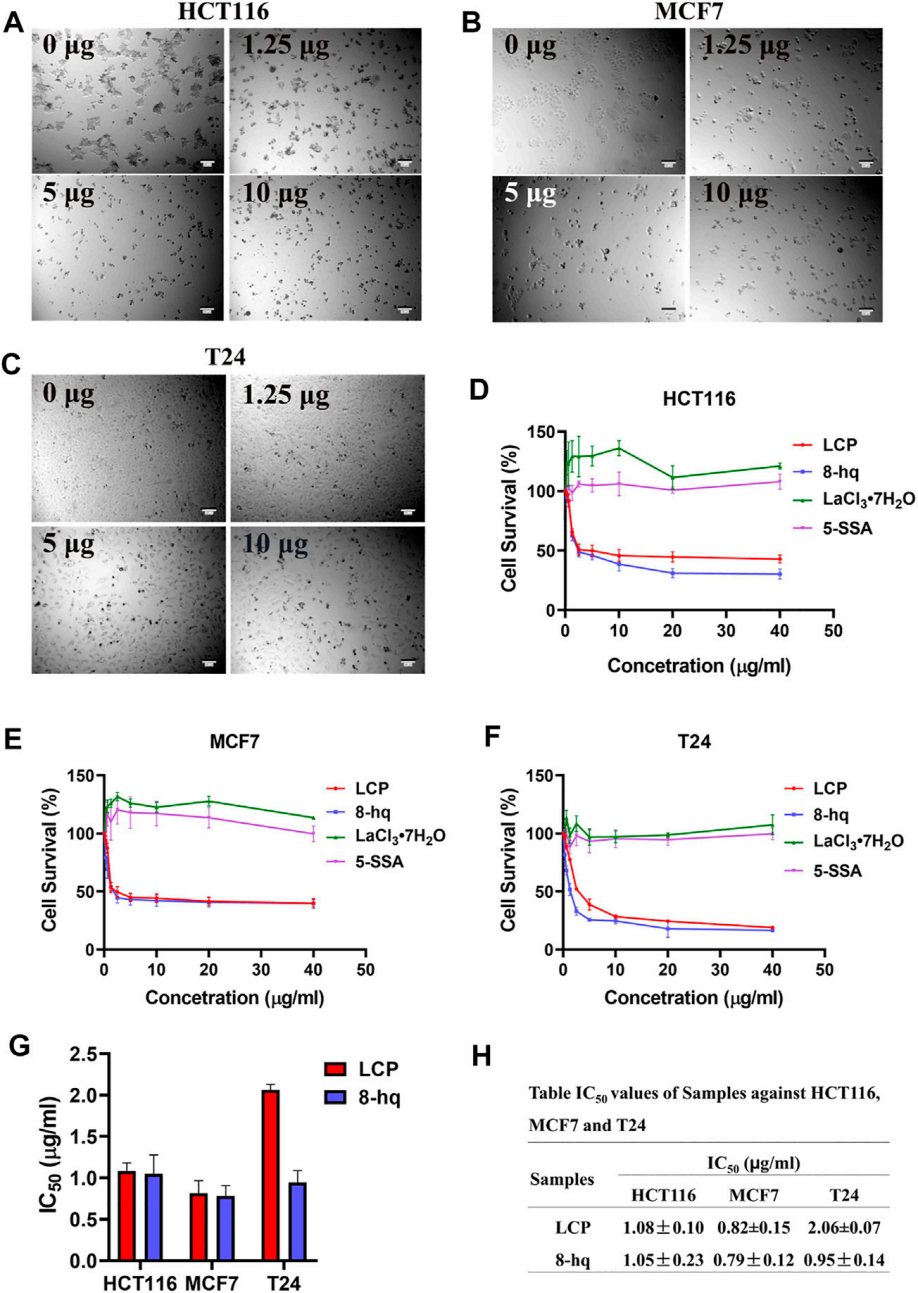
Tumor cell survival rate of complex and ligands. The tumor cell morphology under the microscope at different concentration (0 μg/ml, 1.25 μg/ml, 5 μg/ml, and 10 μg/ml) of LCP-1 **(A–C)**, CCK-8 assay showed that the LCP and ligands inhibit the growth of HCT116, MCF7, and T24 cells **(D–F)**, and calculation of IC_50_ of LCP and 8-hq in HCT116, MCF7, and T24 cells **(G,H)**. Data presented as the mean ± SD, *n* = 3.

The suppression effects of LCP-1 and those of three ligands (8-hq, LaCl_3_·7H_2_O, and 5-SSA) toward tumor cells (HCT116, MCF7, and T24) have been further studied. The morphological observation of the three treated tumor cells under the microscope is shown in Figures 7A–C. As shown in the figures, the three tumor cells’ morphology gradually changed with the increase of LCP-1 concentration and then lost their original morphology. The data of the relative inhibition rate of LCP-1 and complexes on the tumor cells are shown in Figures 7D–F. Compared with the two ligands LaCl_3_·7H_2_O and 5-SSA, the LCP-1 and 8-hq possess stronger inhibitory effect against the three kinds of tumor cells, and the inhibition rate increased with the increase in the complex’s concentration with the survival rate of 42.92 ± 3.33 (HCT116), 39.97 ± 3.33 (MCF7), and 18.96 ± 0.85 (T24), while the survival rate of 8-hq is 30.37 ± 4.33%, 39.80 ± 4.34, and 16.60 ± 1.05%. Furthermore, the IC_50_ values of LCP and 8-hq were calculated and summarized in Figures 7G,H, and all the IC_50_ values of 8-hq are lower than those of LCP-1 against the three tumor cells, with the antitumor activities such as MCF7>T24 > HCT116. Although 8-hq shows a relatively strong inhibitory effect in a certain range of concentrations, it possessed toxicity to the human red blood cells and dsDNA (Yan et al., 2015; Duarte et al, 2000). After mixed with the less toxic rare-earth complexes (Chen et al., 2013; Ghosh et al., 2017), the LCP complex obtains antibacterial and antitumor activities with lower toxicity and high stability (Taha et al., 2016), which has the potential to act as antibacterial compounds and antitumor agents.

## Conclusion

The 10-gram-scale ternary lanthanum CPs were synthesized using a facile and manual grind method, with high antibacterial activities. Metal ionic salts react rapidly with good chelating ligands under mechanochemical conditions. The partial raw materials, such as LaCl_3_ and 5-SSA, had low antimicrobial and antitumor effect, while the final products showed appreciable bifunctional effect owing to the synergy effect. This study could be attractive for the development of environmentally friendly processes with large-scale synthesis of CPs and other functional materials, which can be harnessed as bactericides against pathogenic bacteria and medicine for tumor cytotoxicity. In view of its good inhibitory effect on bacteria and tumor cells, we believe that LCP has good developmental and application value in the bacterial pollution control and antitumor treatment.

## Data Availability

The original contributions presented in the study are included in the article/Supplementary Material. Further inquiries can be directed to the corresponding authors.
