# Supplementary material for: Ten-Gram-Scale Mechanochemical Synthesis of Ternary Lanthanum Coordination Polymers for Antibacterial and Antitumor Activities
Source: Front Chem. 2022 Jun 14;10:898324. doi: 10.3389/fchem.2022.898324 (PMC9237552; doi:10.3389/fchem.2022.898324)
Supplement: Supplementary file 1 [file DataSheet1.docx]

**Ten-gram-scale mechanochemical synthesis of ternary lanthanum coordination polymers for antibacterial and** **antitumor** **activities**

Liying Zhang ^a^, Haoran Shi ^a^, Xiao Tan ^b^, Zhenqi Jiang ^c,^ ^[[1]](#footnote-1)^*, Ping Wang ^a, b,^ ^[[2]](#footnote-2)^*, Jieling Qin ^a,b,^ ^[[3]](#footnote-3)^*

^a^ School of Life Sciences and Medicine, Shandong University of Technology, Zibo 255049, China

^b^ Tongji University Cancer Center, Shanghai Tenth People's Hospital, School of Medicine, Tongji University, Shanghai 200092, China

^c^ Institute of Engineering Medicine, Beijing Institute of Technology, Beijing 100081, China

* Correspondence: [7520200073@bit.edu.cn](mailto:7520200073@bit.edu.cn) (Zhenqi Jiang), pwang@sdut.edu.cn (Ping Wang) and qinjieling770@ hotmail.com (Jieling Qin)

**1. Materials**

Lanthanide chloride hydrate (LaCl_3_·7H_2_O) was prepared by dissolving lanthanide oxide (La_2_O_3_, 99.99%, Shanghai Yuelong, China) in 6 M HCl, followed by vaporization in a vacuum oven to yield a light, solid white sample. 5-Sulfosalicylic acid sodium salt (5-SSA, AR) and 8-hq (8-Hydroxyquinoline, AR) were purchased from Sinopharm Chemical Reagent Co., Ltd. and used as received without further purification.

The inocula (*Escherichia coli, Staphylococcus aureus, Salmonella typhi*, and *Pseudomonas aeruginosa*) were obtained from the original bacterium (American type culture collection, ATCC) using a microbiological loop. After shaking using a vortex mixer, bacteria were cultured in a thermostatic water shaker for 24 h (*S. aureus* culture requires 48 h).

**2. Characterization**

Elemental analyses of ternary lanthanide CPs were carried out using a Flash 111A elemental analyzer. UV-Vis spectra were recorded across an absorbance range of 200 to 600 nm using a Shimadzu UV2450 spectrometer. FT-IR spectra were recorded using a Nicolet Nexus 470 IR spectrometer within the 4000-400 cm^−1^ wavenumber range using the KBr pellet technique. X-ray diffraction (XRD) patterns were collected using a Bruker AXS D8 Superspeed X-ray diffractometer through a Ni-filtered Cu Kα radiation (40 KV, 250 mA). The acquired products were further examined using a field-emission scanning electron microscope (FESEM, JEOL, JSM-7001F) and transmission electron microscopy (TEM, JEOL, JEM-2100). The elemental analysis was analyzed using Elementar Vario MACRO cube VSM SQUID-VSM. The ICP-MS and ICP-OES were recording using Agilent 7500 and Varian 710-ES. Low resolution electrospray ionization mass spectrometry LRMS (ESI-MS) experiments were carried out in negative mode with Agilent Technologies LC/MSD Trap SL AGILENT instrument (mobile phase Acetonitrile) with the following settings: Nebulizer = 20 psi, Dry gas = 5 L/min, Dry Temp = 325°C. The NMR and DLS were using Advance Bruker 400M and Malvern Mastersizer 3000 respectively.

3. **Preparation of** **ternary CPs in solution phase method**

For the synthesis of solution-state CPs, 5-SSA, LaCl_3_·7H_2_O, and 8-hq were first dissolved in distilled water and absolute ethanol separately. Next, the three types of solutions were combined with continuous stirring for 30 min. After that, the appropriate amount of HCl or NaOH was added to adjust the pH to 6.8. The color changed suddenly from a bright yellow to tea-green at a pH of 6.8. The resulting complex was continuously stirred at 25 ℃. After 12 h, the samples were washed with DW/ EtOH three times. Lastly, the obtained CPs were dried in vacuum at 60 ℃ for another 12 h to obtain a yellow, solid product.

**4. Determination of *in vitro* antibacterial activity**

The bacterial liquid (*E. coli, S. aureus, S. typhi,* and *P. aeruginosa*.) was standardized by measuring the absorbance (A 625=0.1; SP-1800 Spectrophotometer, Pye-Unicam, Cambridge, U.K.) and determining the optical density (OD) to ensure a final concentration of approximately 1*10^5^-1*10^7^ CFU/mL.

For the determination of the zone of inhibition, the ternary lanthanide sample (100 μL, DMF as solvent) with the bacterial suspension cultured in nutrient broth (NB) (bacterial concentration ranging from 10^5^-10^7^ CFU/mL) was dropped in an Oxford cup and placed on a nutrient agar plate (Müller-Hinton agar or Sabouraud Dextrose agar). After incubation at 37°C for 48 h, the inhibition zone on the plate was measured.

MIC indicates the lowest biomaterial concentration that can inhibit the visible growth of bacteria, while MBC represents the minimum concentration of the biomaterial to kill 99.9% of the bacteria. MIC was measured using the broth microdilution method in our study. Each experiment was performed three times to confirm MIC and MBC results. For determining the MIC, gradient concentrations of CPs in the test tubes were each treated with several microbial inocula, prepared in a shaker, and cultured at 37°C for 24 h to detect the extent of inhibition. To detect MBC, an appropriate amount of each culture medium showing no visible growth was further inoculated in agar plates. After aerobic incubation at 37 ℃ for 24 h, the numbers of surviving organisms were determined.

Table S1. The ratios of reactants in solid and solution state

| Sample | LaCl_3_·7H_2_O (g) | 5-SSA (g) | 8-hq (g) | Preparation method |
| --- | --- | --- | --- | --- |
| LCP-1 | 1.857 | 1.271 | 1.452 | Solid state |
| LCP-1 | 18.57 | 12.71 | 14.52 | 10-gram-scale |
| LCP-2 | 1.857 | 2.542 | 0.726 | Solid state |
| LCP-3 | 1.857 | 1.271 | 1.452 | Solution state |


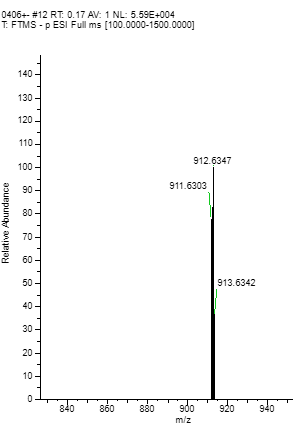


**Figure S1.** Mass spectrum of LCP-1. The sample was dissolved in acetonitrile and carried out in negative mode with mobile phase acetonitrile.


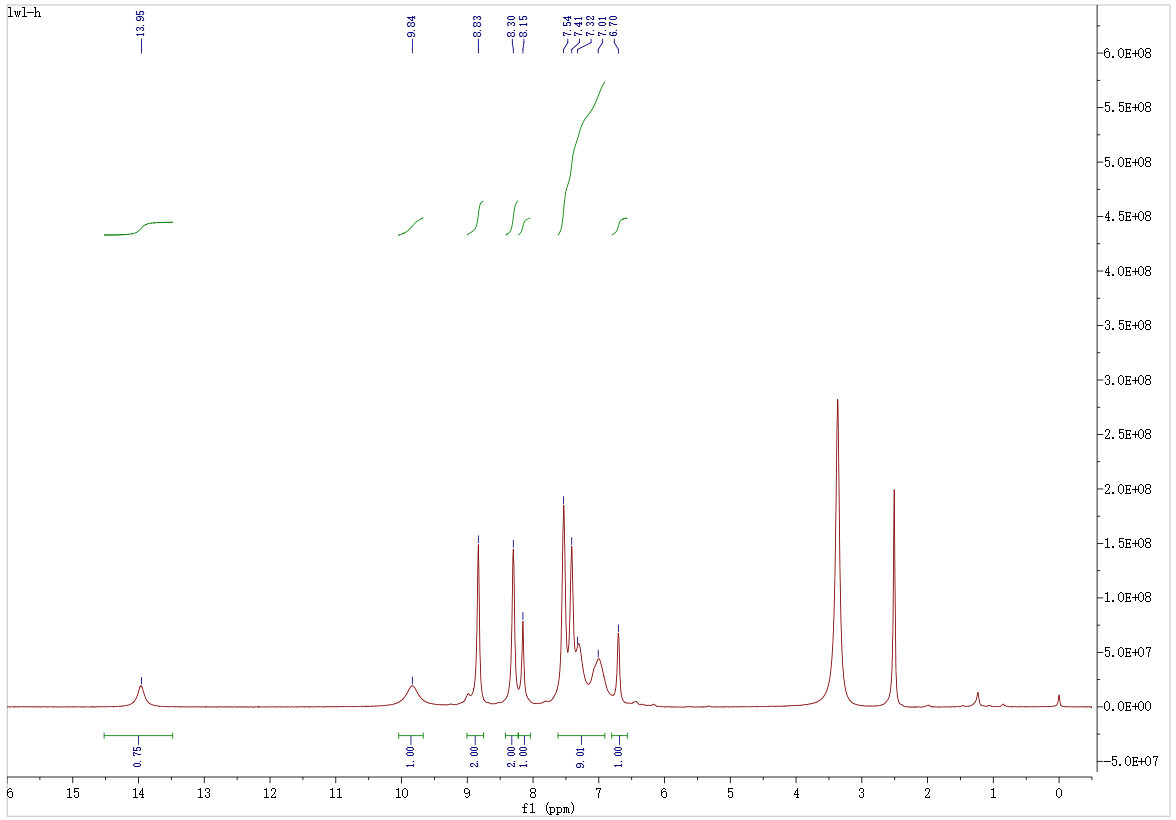


Figure S2. ^1^H-NMR spectra for LCP-1 (Solvent: DMSO).


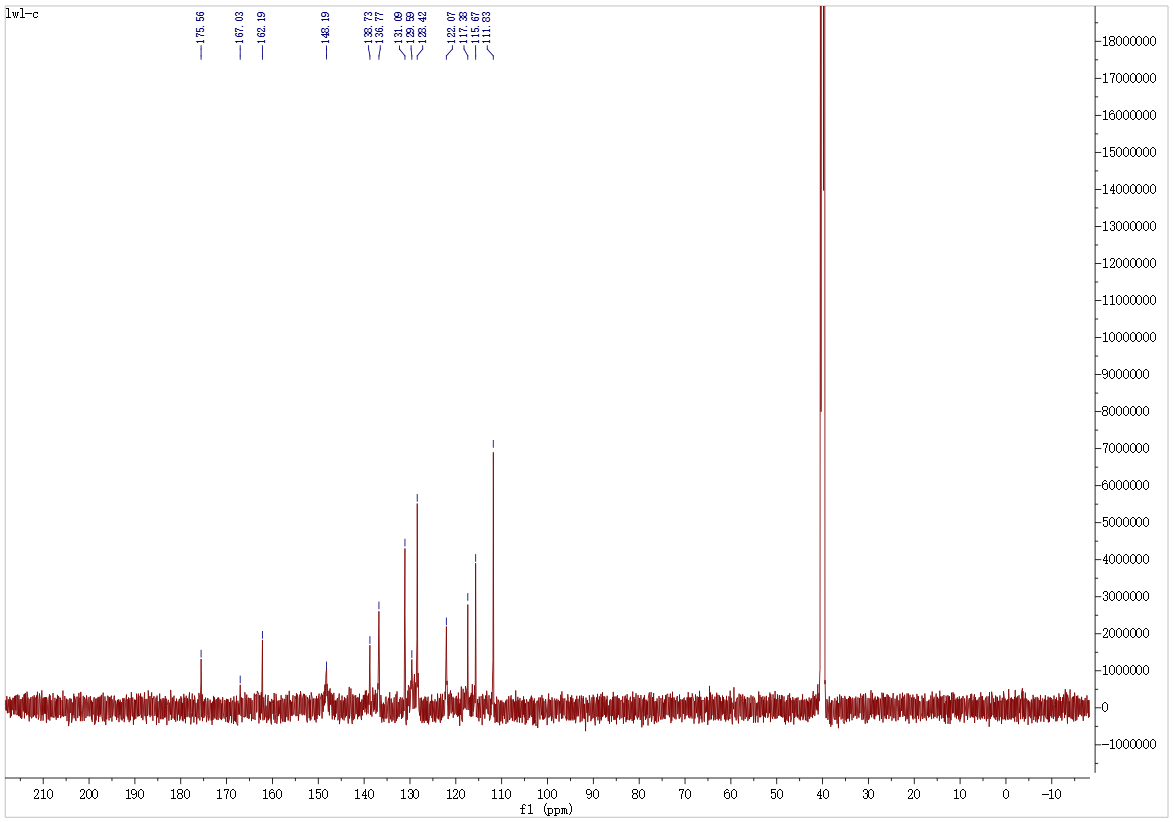


Figure S3. ^13^C-NMR spectra for LCP-1 (Solvent: DMSO).


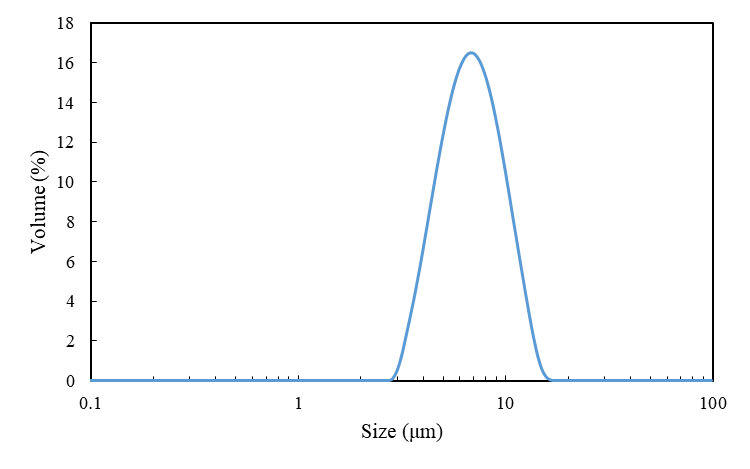


Figure S4. The size distribution of LCP-1 in water


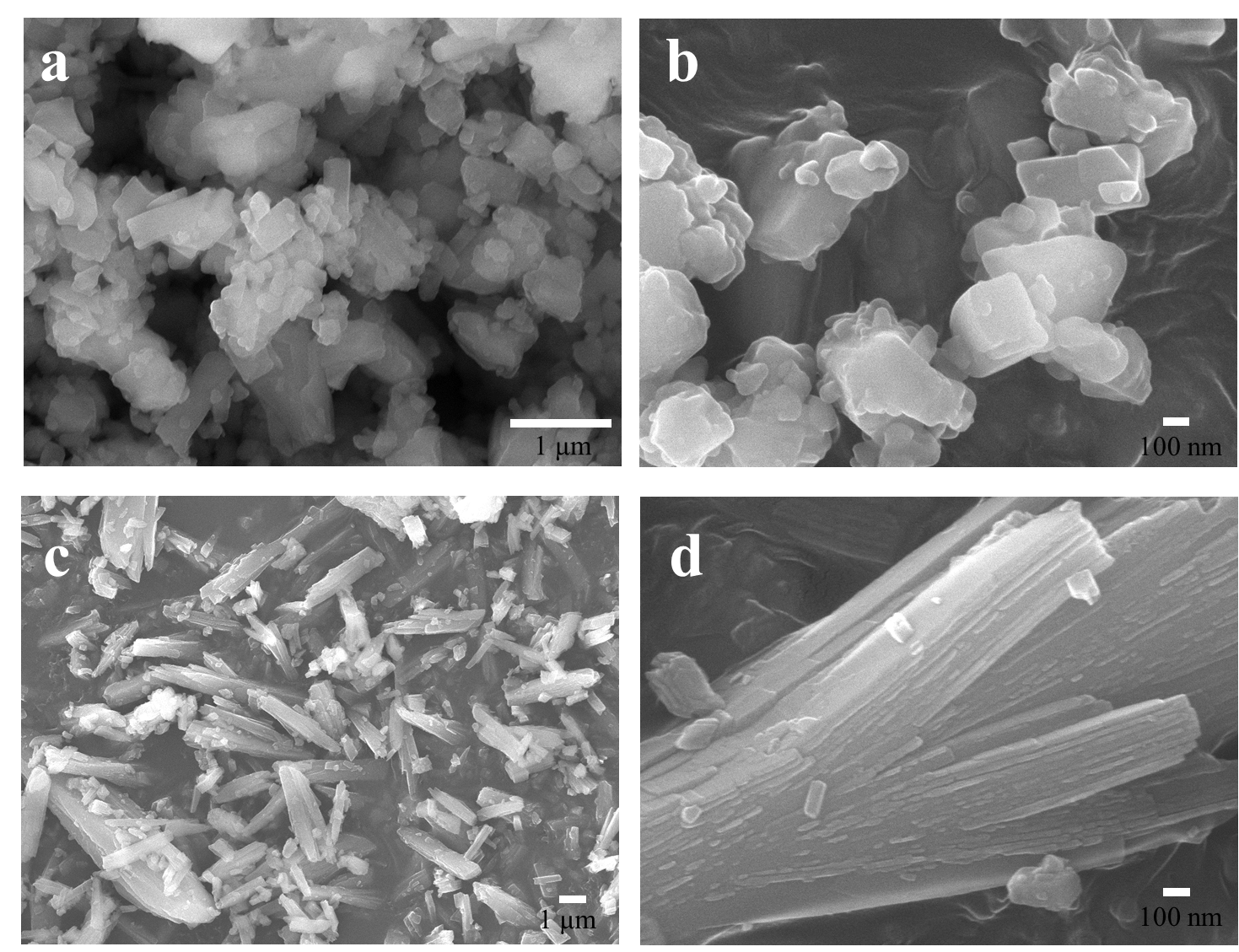


Figure S5. SEM images of (a-b) LCP-2 and (c-d) LCP-3


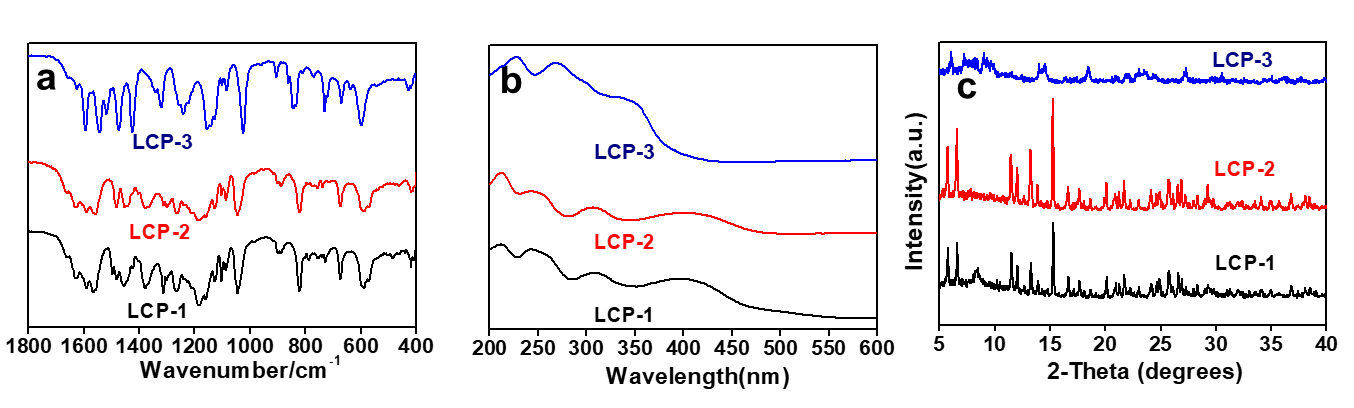


Figure S6. FT-IR (a), UV-Vis (b) and XRD (c) images of LCP-2 and LCP-3

Table S2. FT-IR peaks of product and reactants

| Sample | ν_O-H_ | ν_O-H_ | ν_C-O_ | ν_C=N_ | δ_O-H_ | ν_as_ | ν_s_ | △ν |
| --- | --- | --- | --- | --- | --- | --- | --- | --- |
| S1 | 3300-3700 |  | 1103 | 1591 |  | 1558 | 1313 | 245 |
| 8-hq |  | 3051 | 1093 | 1579 | 1223 |  |  |  |
| 5-SSA | 3200–3500 |  |  |  |  | 1678 | 1430 | 248 |

Table S3. ZOI, MIC and MBC of the composites and their reactants

| Sample | Inhibition zone (mm)/ MIC/ MBC | | | |
| --- | --- | --- | --- | --- |
|  | *E. coli* | *S. aureus* | *S. typhi* | *P. aeruginosa* |
| S2 | 19/200/ 200 | 20/100/ 100 | 20/ 200/ 200 | 18/ 100/ 200 |
| S3 | 20/ 200/ 400 | 21/100/ 200 | 20/ 200/ 400 | 19/ 200/ 200 |





Figure S7. N_2_ adsorption-desorption isotherms of LCP-1


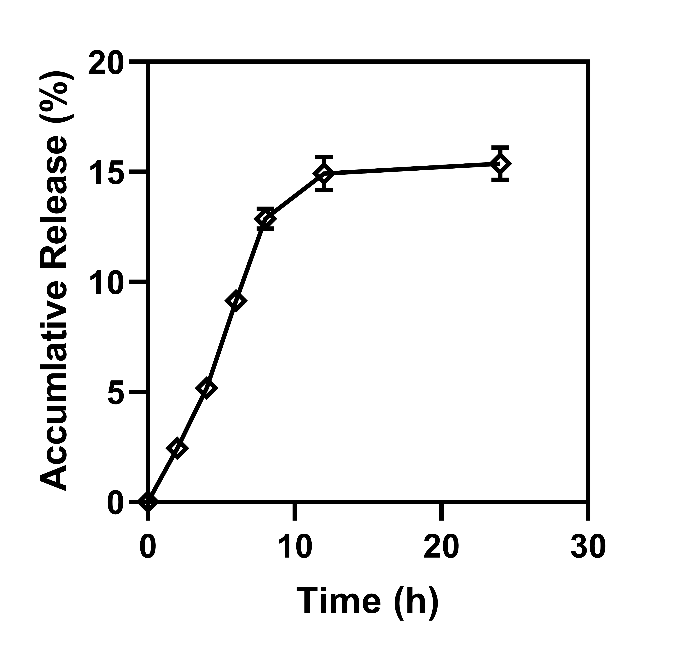


Figure S8. *In vitro* cumulative release of LCP-1 in PBS at pH 7.4 for 24 h at 37 °C. Data represent the mean ± SD (n=3).

1. [↑](#footnote-ref-1)
2. [↑](#footnote-ref-2)
3. [↑](#footnote-ref-3)
